# An Improved Synthesis of Pentacene: Rapid Access to a Benchmark Organic Semiconductor

**DOI:** 10.3390/molecules17044625

**Published:** 2012-04-20

**Authors:** Chandrani Pramanik, Glen P. Miller

**Affiliations:** Department of Chemistry & Materials Science Program, University of New Hampshire, Durham, NH 03824, USA

**Keywords:** pentacene, acene, semiconductor, organic semiconductor, thin-film, organic electronic

## Abstract

Pentacene is an organic semiconductor used in a variety of thin-film organic electronic devices. Although at least six separate syntheses of pentacene are known (two from dihydropentacenes, two from 6,13-pentacenedione and two from 6,13-dihydro-6,13-dihydroxypentacene), none is ideal and several utilize elevated temperatures that may facilitate the oxidation of pentacene as it is produced. Here, we present a fast (~2 min of reaction time), simple, high-yielding (≥90%), low temperature synthesis of pentacene from readily available 6,13-dihydro-6,13-dihydroxypentacene. Further, we discuss the mechanism of this highly efficient reaction. With this improved synthesis, researchers gain rapid, affordable access to high purity pentacene in excellent yield and without the need for a time consuming sublimation.

## 1. Introduction

Pentacene is a benchmark organic semiconductor in thin-film organic electronic devices due to its *π*-conjugated electronic structure, its relatively low HOMO-LUMO gap and the relatively high charge carrier mobility of its solid state films. It is the most common p-type organic semiconductor in OFET devices [1,2,3,4]. Extensive efforts have also been carried out to integrate pentacene into OLED [5,6] and photovoltaic [7,8,9] devices.

**Scheme 1:**
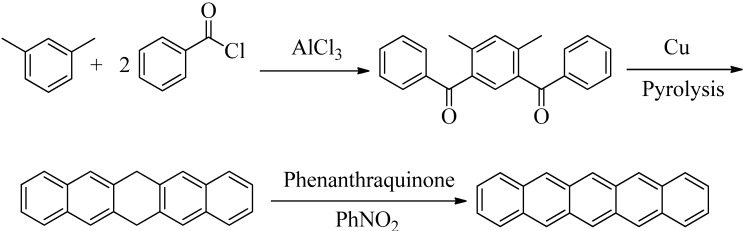
The first synthesis of pentacene by Clar [10,11].

Pentacene is a deep blue, crystalline material of high reactivity that shows only sparing solubility in organic solvents. Unlike anthracene or tetracene, pentacene cannot be isolated from petroleum; hence it needs to be synthesized. The first synthesis of pentacene was accomplished by Clar in 1929 via a transfer dehydrogenation of 6,13-dihydropentacene using phenanthraquinone [10,11]. Thus, successive Friedel-Craft acylations of *m*-xylene using benzoyl chloride produces 1,3-dibenzoyl-4,6-dimethylbenzene. With further heating in the presence of copper, the diketone is transformed to 6,13-dihydropentacene. Transfer dehydrogenation in the presence of phenanthraquinone and nitrobenzene yields pentacene in unreported yield (Scheme 1).

**Scheme 2:**
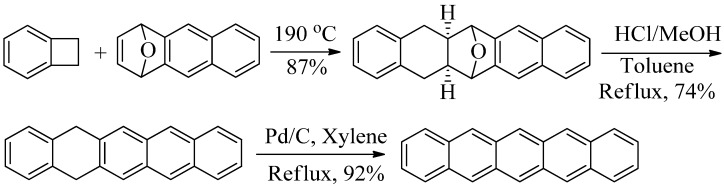
Synthesis of pentacene from 5,14-dihydropentacene via a Pd/C dehydrogenation [12].

Similarly, pentacene has also been synthesized from 5,14-dihydropentacene via a transition metal catalyzed dehydrogenation [12]. Thus, Hart reacted benzocyclobutene, an *o*-quinodimethane precursor, with anthracene-1,4-endoxide (prepared in a multi-step synthesis involving 2,3-dihydronaphthalene and furan) to produce a dihydropentacene hydrate that was dehydrated using HCl. A subsequent Pd/C catalyzed dehydrogenation produced pentacene (59% overall yield from anthracene-1,4-endoxide, Scheme 2).

**Scheme 3:**
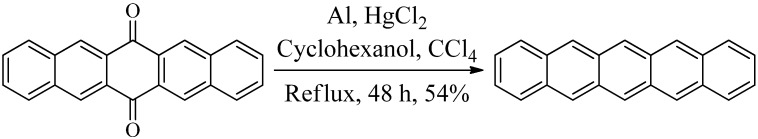
Synthesis of pentacene from pentacene-6,13-dione using a Hg-Al amalgam [16].

A third synthetic pathway to pentacene involves reduction of pentacene-6,13-dione which is itself synthesized via four successive aldol condensations involving two equivalents of *o*-phthalaldehyde and one equivalent of 1,4-cyclohexanedione [13,14]. The reduction of pentacene-6,13-dione using either Al in cyclohexanol (5–51% yield) [15] or an Al/HgCl_2 _mixture in cyclohexanol/carbon tetrachloride (54% yield) [16] requires multiple days to produce pentacene (Scheme 3). The high boiling solvent conditions may hasten pentacene degradation as it is produced. Moreover, CCl_4_ is a regulated chemical and HgCl_2_ is highly toxic.

**Scheme 4:**
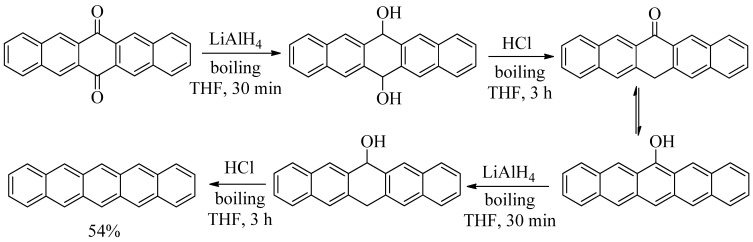
Synthesis of pentacene from pentacene-6,13-dione using successive LiAlH_4_ reductions followed by acid dehydrations [17].

A fourth procedure to prepare pentacene involves the reaction of pentacene-6,13-dione in dry, boiling THF with LiAlH_4_ for 30 min followed by the addition of 6 M HCl and three additional hours of boiling. This procedure yields a crude product mixture that reportedly includes pentacen-6(13*H*)-one and its tautomer 6-hydroxypentacene. Following filtration and washing, the crude product is subjected to the same reaction sequence a second time to produce pentacene in 54% overall yield [17] (Scheme 4).

**Scheme 5:**
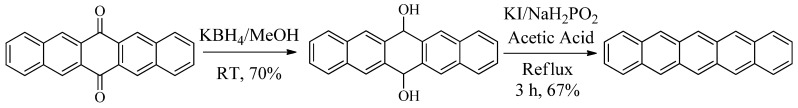
Synthesis of pentacene from pentacene-6,13-dihydro-6,13-diol using potassium iodide, sodium hypophosphite and acetic acid [18].

A fifth procedure to produce pentacene involves the reduction of 6,13-dihydro-6,13-dihydroxypentacene using potassium iodide and sodium hyposulfite in boiling acetic acid for three hours (Scheme 5). In this way, pentacene is produced in 67% yield [18].

Finally, a sixth procedure [19] involves a minimum of 30 minutes of stirring of 6,13-dihydro-6,13-dihydroxypentacene and SnCl_2_ (1:2 mole ratio) in acetone. The resulting crude pentacene was isolated by centrifuge, and washed with both methanol and THF. Purification by sublimation resulted in pure pentacene in 60% overall yield from 6,13-dihydro-6,13-dihydroxypentacene. 

This latter procedure is closest to the improved synthesis of pentacene reported here. However, the improved synthesis utilizes HCl as co-reagent, involves shorter reaction times, does not require a sublimation step, and results in a significantly higher overall yield.

## 2. Results and Discussion

**Scheme 6:**
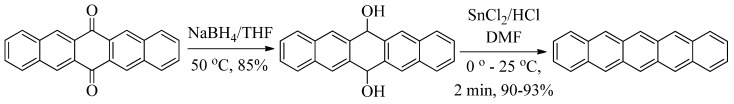
Synthesis of pentacene from 6,13-dihydro-6,13-dihydroxypentacene using SnCl_2_/HCl/DMF.

**Figure 1:**
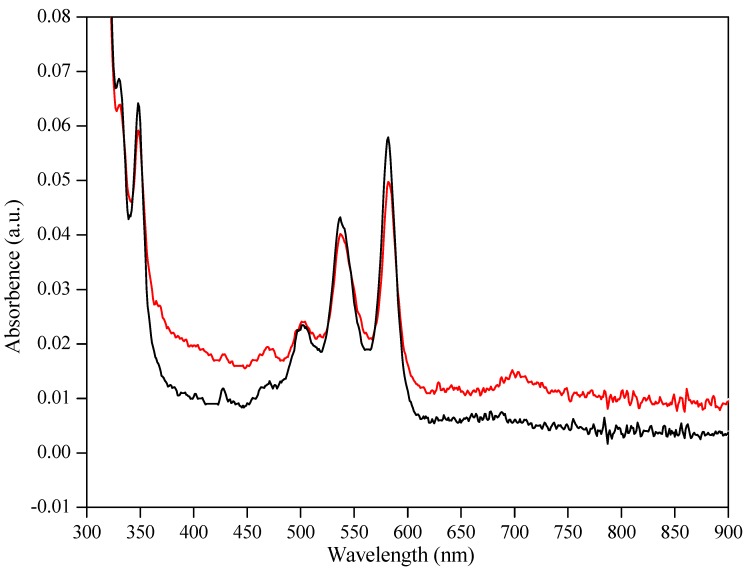
UV-Vis comparison of crude product prepared as described (black line)*versus* a commercial pentacene sample (red line) that has been purified by sublimation (both samples prepared in *o*-dichlorobenzene solvent).

Here, we present a simple, fast, high-yielding (≥90%), scalable synthesis of pentacene under mild conditions. Thus, 6,13-dihydro-6,13-dihydroxypentacene, readily prepared from pentacene-6,13-dione, is reduced to pentacene at low temperature using SnCl_2_ and HCl in an appropriate solvent, preferably DMF or acetone. The low temperature reaction is high yielding (e.g., 90–93% in DMF, Scheme 6) and is complete within 1–2 min. The reaction begins working within seconds at 0 °C, but it is more convenient to perform at or near RT. The work-up involves only simple filtration and washing with water, acetone and hexane. The crude reaction product is high purity pentacene, a slightly soluble blue solid that exhibits the expected molecular ion [*m/z* 278 by MALDI-MS (S_8_ matrix) and LDI-MS] and UV-Vis λ_max_ values [*o*-dichlorobenzene (ODCB) solvent: 501, 537, 582 nm], both in agreement with the literature [17,20]. Most important, there is no mass spectrometry or UV-Vis evidence for pentacene-6,13-dione in the reaction product, a consequence of the mild reaction conditions, short reaction time, and simple work-up. In fact, the crude product prepared in this manner is identical to a commercial sample purified by sublimation (TCI America, P0030) as determined by UV-Vis analysis (Figure 1).

The use of HCl and the choice of solvent are both key to the success of the reaction. Thus, the SnCl_2_ reduction of 6,13-dihydro-6,13-dihydroxypentacene is considerably faster in the presence of HCl, regardless of choice of solvent. All eight solvents tested (*i.e.*, DMF, DMSO, THF, dioxane, acetone, CH_3_CN, AcOH, MeOH) gave pentacene product but the rates and yields of reactions varied as a function of solvent. Of these solvents, acetone and DMF gave very fast reactions (1–2 min) with high yields (≥90%). In a typical synthesis, 6,13-dihydro-6,13-dihydroxypentacene is first dissolved in appropriate solvent along with SnCl_2_. Concentrated HCl is added leading to the instant formation of blue pentacene, most of which immediately precipitates from solution. In acetone, a small amount of pentacene is observed to form even before the HCl addition. In DMF, pentacene only forms after the addition of HCl. The HCl addition is exothermic, especially in DMF. Thus, if the reaction is run on a relatively large scale (e.g., ≥1 g), then the reaction vessel should be submerged in an ice-water bath. For reactions run on a smaller scale, the reaction vessel can simply be submerged in a water bath held at room temperature.

Because 6,13-dihydro-6,13-dihydroxypentacene and SnCl_2_ are both highly soluble in DMF, this solvent is particularly appropriate for larger scale reactions. Thus, after dissolving 1.0 g of 6,13-dihydro-6,13-dihydroxypentacene and 1.5 g of SnCl_2_ in 13 mL of DMF, 20 mL of concentrated HCl was added over one minute with stirring while the reaction vessel was submerged in an ice water bath at 0 °C. Following work-up, 0.83 g of pure pentacene was isolated (93% yield). We see no reason why the DMF reaction could not be scaled several additional orders of magnitude, although precautions would need to be taken to properly manage the heat associated with mixing larger quantities of DMF and HCl (e.g., by submerging the reaction vessel in an ice-water bath).

**Scheme 7:**
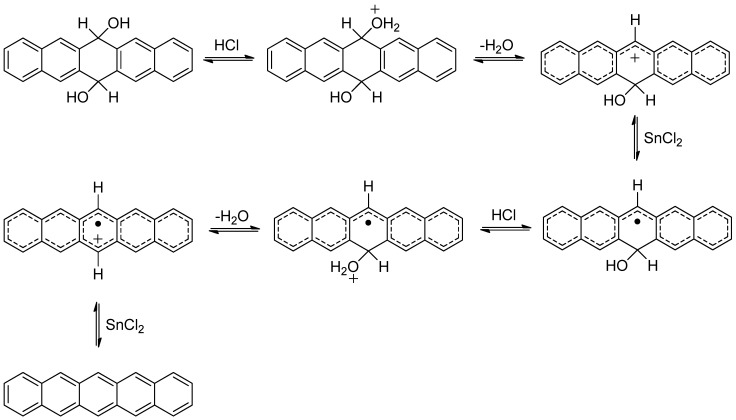
Proposed mechanism for rapid formation of pentacene using a combination of HCl and SnCl_2_.

We propose that the use of concentrated HCl facilitates the conversion of 6,13-dihydro-6,13-dihydroxypentacene to pentacene via protonation of the hydroxyl groups followed by elimination of water and formation of short-lived, electron deficient carbocations that act as electron acceptors (Scheme 7). It is also possible that HCl converts SnCl_2_ into a better electron donor species like, for example, SnCl_4_^2−^ or SnCl_6_^4−^. Additionally, we observe that pentacene precipitates much more rapidly from solutions that contain HCl as opposed to those that do not. This is critical in that since solution phase pentacene oxidizes much more rapidly than solid state pentacene. Thus, the use of HCl minimizes the formation of a common oxidation by-product, pentacene-6,13-dione, and eliminates the need to perform a rigorous, time consuming separation like a sublimation.

## 3. Experimental

### 3.1. General Experimental Methods

Reactions were performed in standard glassware except where noted. Mass spectra were recorded on a Shimadzu Kratos Axima-CFR laser desorption ionization (LDI) time of flight (TOF) mass spectrometer (Kyoto, Japan). UV-visible spectra were obtained on a Nikolet Evolution 300 spectrometer (Cambridge, UK) using 1 cm quartz cells. Saturated solutions of pentacene were prepared using spectroscopic grade *o*-dichlorobenzene. NMR spectra were recorded using Varian Mercury 400 MHz (Palo Alto, CA, USA) and Varian Unity INOVA 500 MHz spectrometers (Palo Alto, CA, USA). Chemicals were used as purchased without further purification: 1,4-cyclohexanedione (Aldrich), o-phthalaldehyde (TCI America), NaOH (Alfa Aesar), NaBH_4_ (Sigma-Aldrich), SnCl_2_ (Sigma-Aldrich), EtOH (Pharmaco-AAPER), THF (EMD Chemicals), CHCl_3_ (Fisher Scientific), *o*-dichlorobenzene (Acros Organics), concentrated HCl (EMD Chemicals).

*Pentacene-6*,*13-dione*. Aqueous NaOH (10%, 5.96 g, 149 mmol) was slowly added to a solution of *o*-phthalaldehyde (10 g, 74.6 mmol) and 1,4-cyclohexanedione (4.18 g, 37.3 mmol) in ethanol (460 mL) under a N_2_ atmosphere. The solution turned from yellow to golden brown to dark brown before a yellow solid corresponding to pentacene-6,13-dione precipitated. After stirring the reaction mixture for four hours, the crude reaction mixture was filtered and washed with ethanol, water, and methanol until the washings were colorless. The solid residue was dried under vacuum to obtain 11.02 g (96% yield) of bright yellow pentacene-6,13-dione. ^1^H-NMR (500 MHz, CDCl_3_) δ (ppm): 8.96 (s, 4H), 8.14 (m, 4H), 7.72 (m, 4H); ^13^C-NMR (100 MHz, CDCl_3_): δ (ppm): 183.21, 135.45, 130.78, 130.31, 129.99, 129.67; LDI-MS *m*/*z*: [M^+^+O], 324, [M^+^+1] 309, [M^+^] 308.

*6*,*13-Dihydroxy-6*,*13-dihydropentacene.* Solid NaBH_4_ (12.3 g, 324.4 mmol) was slowly added to a 1,000 mL round bottom flask containing a suspension of pentacene-6,13-dione (5 g, 16.2 mmol) in THF (600 mL) at 0 °C. After the addition of NaBH_4_ was complete, the reaction vessel was purged with N_2_ and water (20 mL) was added. The reaction mixture was heated to 50 to 60 °C until homogeneous. After two hours of heating, THF was evaporated at reduced pressure, water was added, and the reaction mixture was filtered. The solids were washed with copious amounts of water followed by a small amount of cold CHCl_3_. After drying, 4.31 g of a white solid was recovered (85% yield) consisting of only one diastereomer of 6,13-dihydroxy-6,13-dihydropentacene. ^1^H-NMR (500 MHz, DMSO-d_6_): δ (ppm): 8.12 (s, 4H), 7.95 (m, 4H), 7.48 (m, 4H), 6.63 (s, 2H), 5.81 (s, 2H). ^13^C-NMR (125.68 MHz, DMSO-*d_6_*): δ (ppm): 138.19, 131.67, 127.47, 125.44, 120.91, 66.98. LDI-MS *m/z*: 312[M^+^], 295 [M^+^-OH], 278 [M^+^-2(OH)].

*Pentacene*. To a N_2_ purged 200 mL round bottom flask was added 6,13-dihydroxy-6,13-dihydropentacene (1 g, 3.2 mmol), SnCl_2_ (1.44 g, 6.4 mmol) and DMF (15 mL). Twenty mL of concentrated HCl was added over ~60 seconds with stirring at 0 °C (ice-water bath) in the dark. The resulting blue reaction mixture was stirred briefly (~15 s) at 0 °C before an additional 20 mL of concentrated HCl was added over ~30 seconds followed by the rapid addition of 100 mL water. The deep blue solids were filtered and washed with copious amounts of water followed by acetone and finally hexane. All washings were performed in air in the dark. The blue solid was dried under reduced pressure yielding 0.83 g of pentacene (93% yield). LDI-MS *m/z*: 278[M+]. UV-Vis (ODCB): λ_max_ (nm): 501, 537, 582; (CHCl_3_) λ_max_ (nm): 497, 533, 577.

## 4. Conclusions

We have demonstrated a simple, fast, high-yielding, scalable, low temperature synthesis of pentacene from 6,13-dihydro-6,13-dihydroxypentacene. Following a simple work-up, crude product is isolated and shown by mass spectrometry and UV-Vis spectroscopy to be of equal quality to commercial samples that have been purified by sublimation. With this synthesis, researchers gain rapid, affordable access to high purity pentacene in excellent yield and without the need for a time consuming sublimation.

## Supplementary Materials

Supplementary materials can be accessed at: http://www.mdpi.com/1420-3049/17/4625/s1.
