# Supplementary material for: An Improved Synthesis of Pentacene: Rapid Access to a Benchmark Organic Semiconductor
Source: Molecules. 2012 Apr 20;17(4):4625–33. doi: 10.3390/molecules17044625 (PMC6268729; doi:10.3390/molecules17044625)

Supporting Information

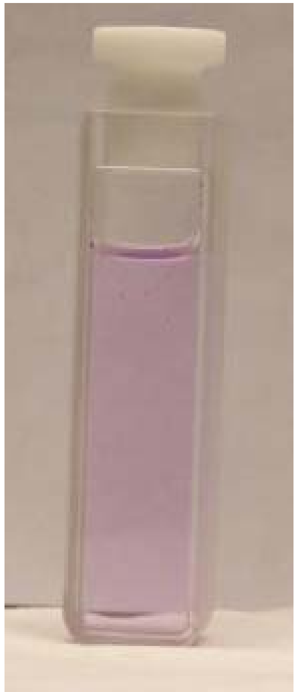

An Improved Synthesis of Pentacene: Rapid Access to a Benchmark Organic Semiconductor

Chandrani Pramanik and Glen P. Miller*

Department of Chemistry & Materials Science Program, University of New Hampshire Durham, NH 03824 -3598 , USA;
E-Mail: glen.miller@unh.edu (G.P.M)

MALDI MS of pentacene as prepared using S_8 as matrix

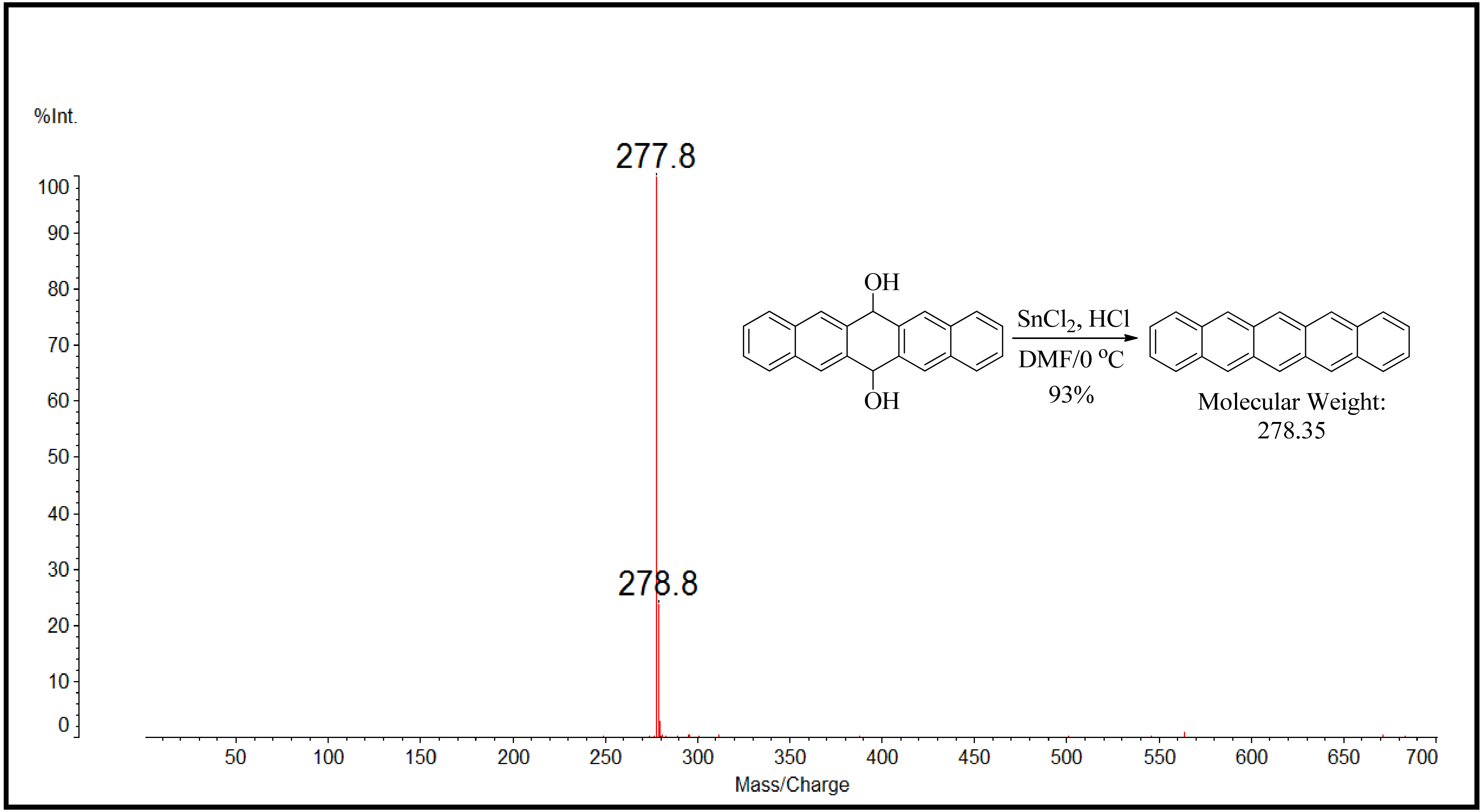

LDI MS of pentacene as prepared

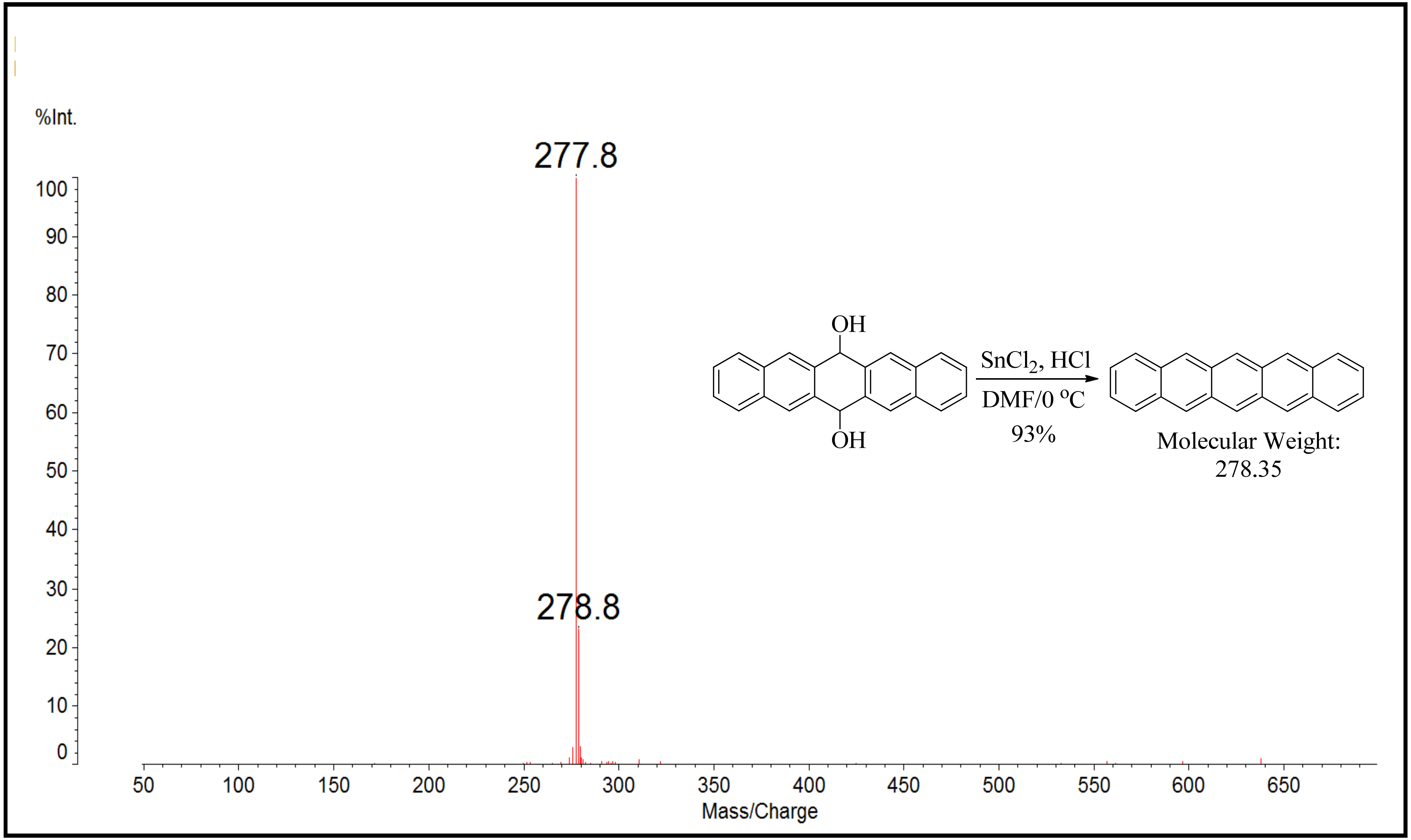

UV-vis spectrum of pentacene as prepared in ODCB

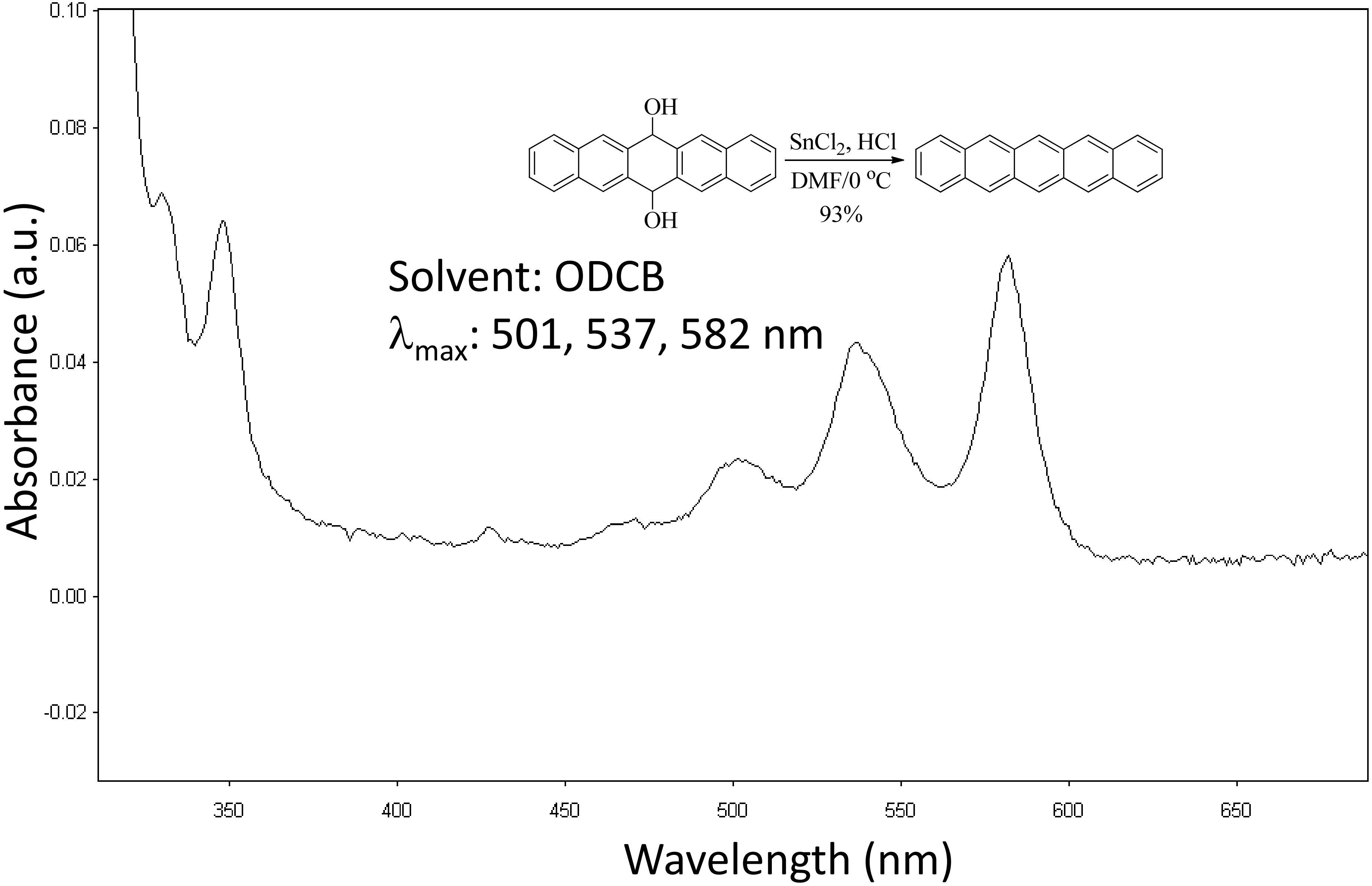

UV-vis spectrum of pentacene as prepared (black) *vs.* commercial pentacene purified by sublimation (red)

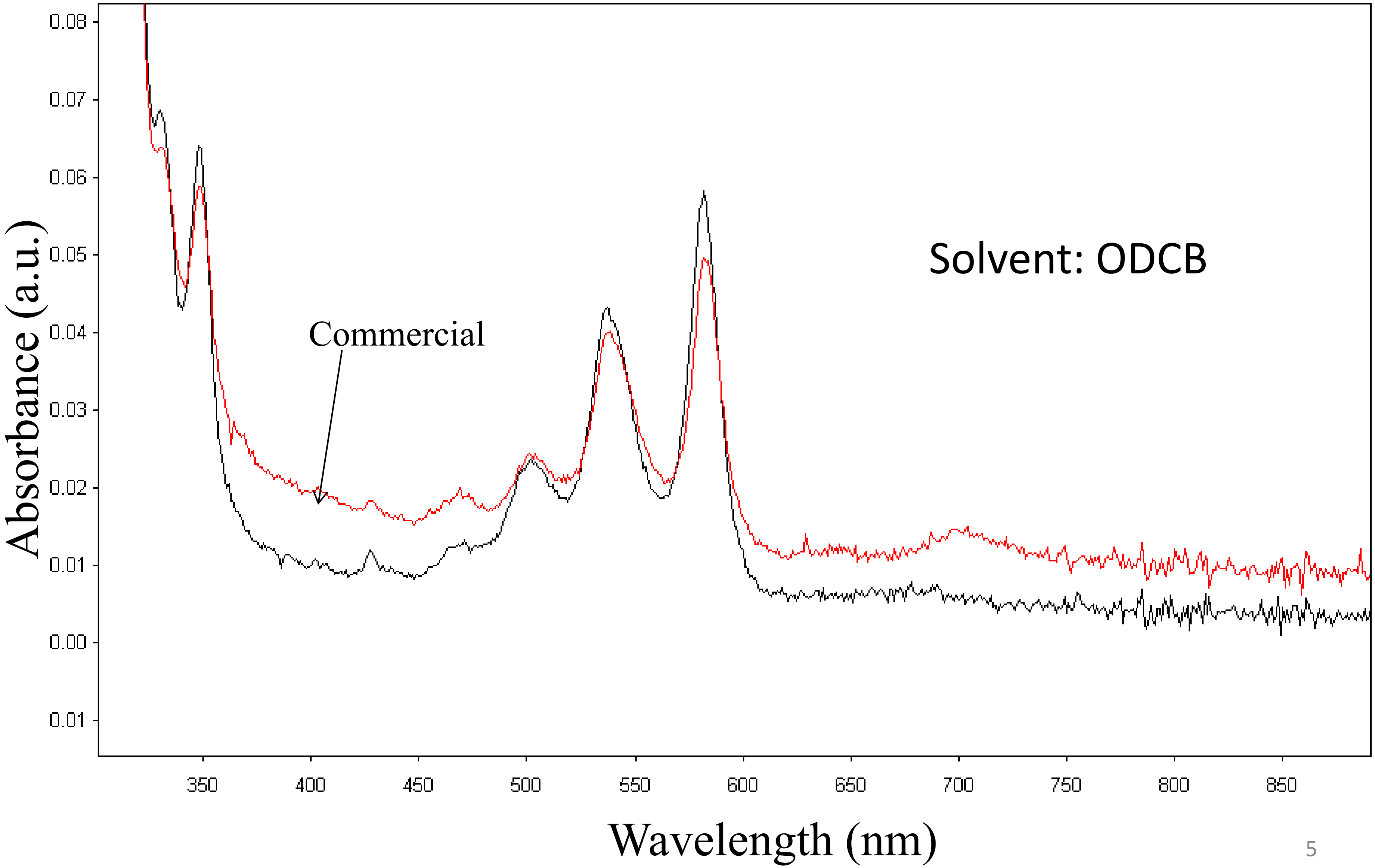

UV-vis spectrum of pentacene as prepared in $CHCl_3$

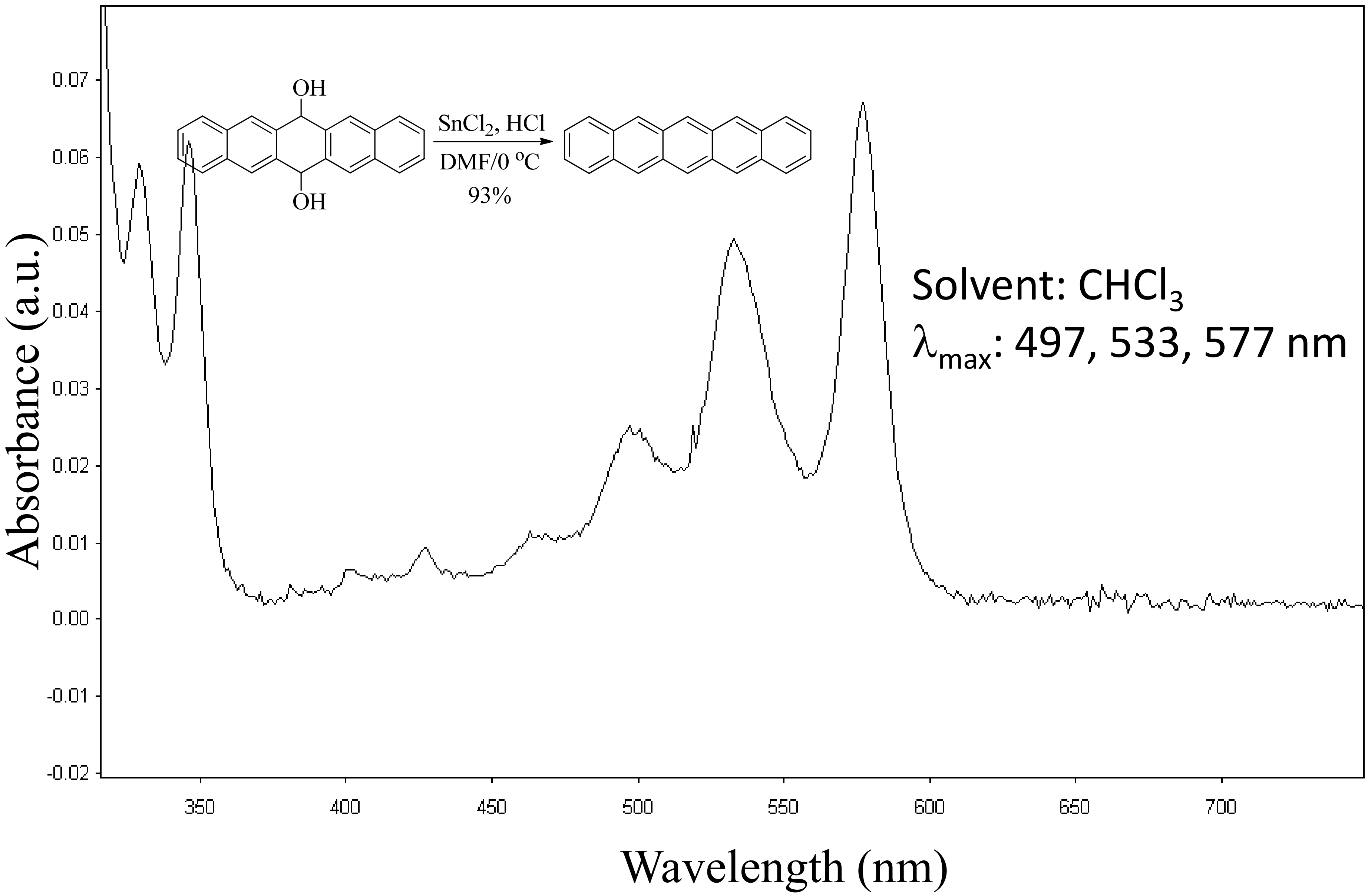

UV-vis spectrum of pentacene as prepared (black) *vs.* commercial pentacene purified by sublimation (red)

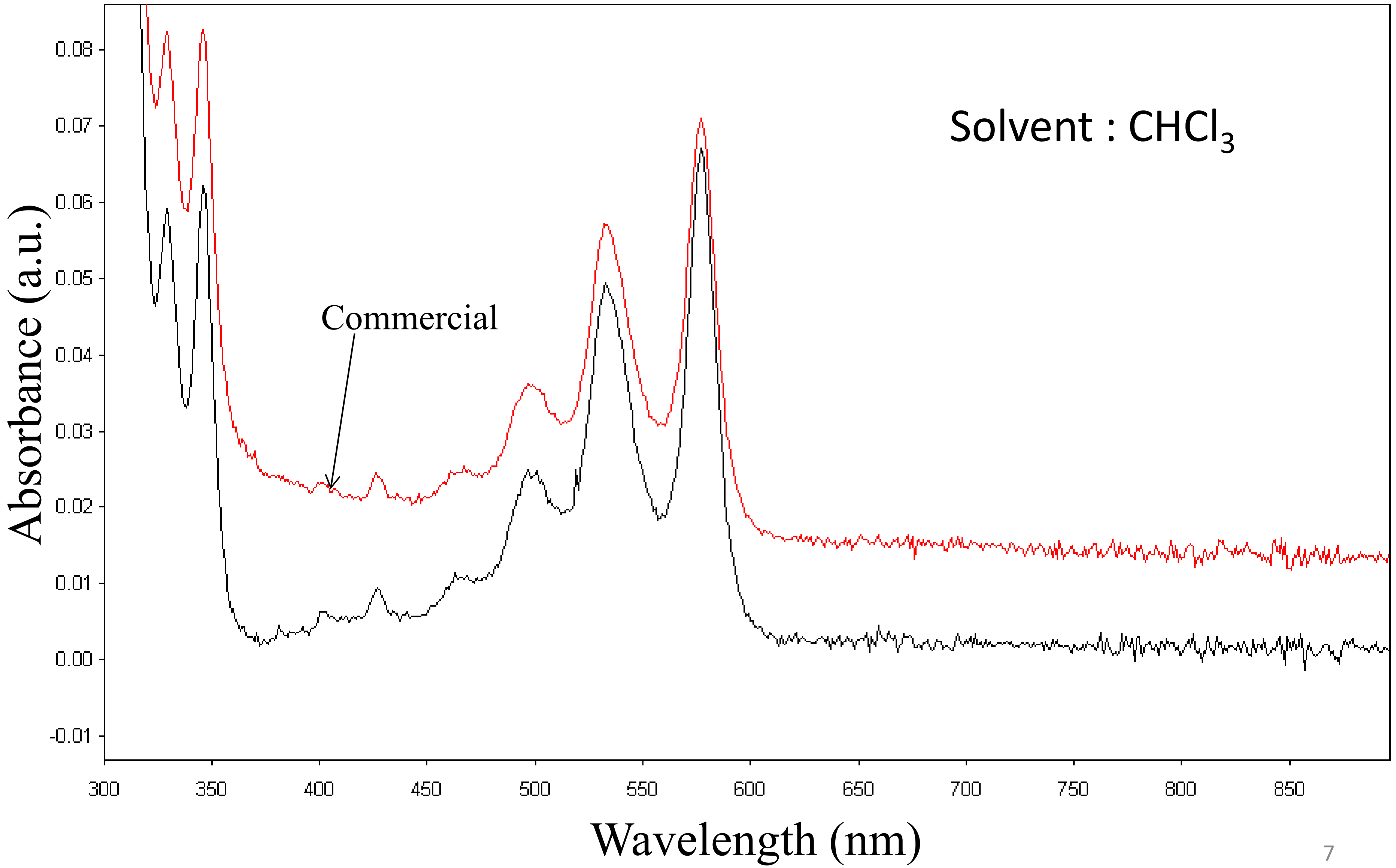

Supplement: Supplementary file 1 [file molecules-17-04625-s001.pdf]
